# Retraction: Cytokinins: a genetic target for increasing yield potential in the CRISPR era

**DOI:** 10.3389/fgene.2026.1875909

**Published:** 2026-05-15

**Authors:** Frontiers Editorial Office

The Journal retracts the 26 April 2022 article cited above.

Following publication, the publisher found evidence of peer review manipulation. As the scientific integrity of the article cannot be guaranteed, and in adherence to the recommendations of the Committee on Publication Ethics (COPE), the article is retracted.

This retraction was approved by the Chief Executive Editor of Frontiers. The authors do not agree to this retraction.

Frontiers would like to thank Elisabeth Bik and the users on PubPeer for bringing the published article to our attention.

